# Editorial: Acute leukemias: molecular characterization, leukemia-initiating cells, and influence of the microenvironment, volume II

**DOI:** 10.3389/fonc.2024.1542306

**Published:** 2025-01-06

**Authors:** Lokman Varisli, Garrett M. Dancik, John A. Copland, Spiros A. Vlahopoulos

**Affiliations:** ^1^ Department of Molecular Biology and Genetic, Faculty of Science, Dicle University, Diyarbakir, Türkiye; ^2^ Department of Computer Science, Eastern Connecticut State University, Willimantic, CT, United States; ^3^ Department of Cancer Biology, Mayo Clinic, Jacksonville, FL, United States; ^4^ First Department of Pediatrics, National and Kapodistrian University of Athens, Athens, Greece

**Keywords:** acute leukemias, leukemia stem cells, tissue microenvironment, immunity, inflammation, homeostasis, stress response

Acute leukemia is a wide group of hematologic malignancies that arise from leukemia-initiating cells (LICs), also known as leukemic stem cells (LSCs), which originate from transformed hematopoietic stem cells (HSCs) in the bone marrow. Leukemic cells accumulate various genetic and epigenetic defects, both inherited from the LICs they originate from and acquired later. Genetic defects in leukemic cells determine their biological behavior and, as expected, affect the prognosis of the disease and its response to treatment. Therefore, molecular characterization of the disease is important for all stages of the disease, such as predicting prognosis, determining the treatment approach, and evaluating the possibility of recurrence. In this context, a better understanding of the molecular mechanisms of acute leukemia will enable the emergence of simpler and more effective treatment approaches and increase the rate of treated patients.

We believe that the studies published in this research topic will contribute to a better understanding of the molecular mechanisms of acute leukemias.

Lin et al., retrospectively analyzed the data of pediatric B-ALL patients from Zhujiang Hospital and identified differential CRLF2 expression levels among molecular subtypes. They also proposed a new cut-off value for CRLF2 overexpression associated with molecular types of pediatric B-ALL and determined that patients with higher CRLF2 expression had a poor prognosis. The prognoses of pediatric B-ALL patients from Zhujiang Hospital, which have different CRLF2 expression levels, were validated using the B-ALL patient cohort in the TARGET database. Finally, the authors proposed that B-ALL patients with poor prognosis could be rapidly identified based on the CRLF2 expression cutoff values they determined.

Xu et al., used various multi-layered molecular approaches to better understand the survival mechanisms of TKI resistant BLASTs in AML using various molecular approaches. They demonstrated that CD44+ and pBAD+ cells required intrinsic homeostatic adaptation to promote BLAST survival and relapse. Finally, the authors proposed that inhibition of TKI-activated compensation mechanisms and disruption of homeostatic adaptation may be a novel treatment approach for patients with FLT3^mut^ AML to prevent relapse.

Li et al., investigated the relationship between ALL-CNS metastasis and neuronal development at gene expression level, aiming to identify possible biomarkers and therapeutic targets in CNS metastasis. The authors identified and analyzed ALL-CNS and neuronal development-related differentially expressed genes (DEGs) by analyzing gene expression datasets deposited in public databases and bioinformatics approaches. In this way, they identified DEGs co-expressed (co-DEGs) with the corresponding predicted miRNAs. Consequently, they specifically identified four co-DEGs (LGALS1, TMEM71, SHISA2, and S100A11) that might link ALL-CNS metastasis and neuronal development, which were validated in CNS-infiltrated AML cells. Finally, the authors proposed that co-DEGs be used as novel biomarkers and therapeutic targets in ALL-CNS metastasis.

Lyu and Lyu, investigated to better understand hyperleukocytosis (HL) in AML since the AML patients with HL generally have a poor prognosis. Therefore, they identified the optimal threshold for HL diagnosis and revealed the molecular mechanisms underlying HL. The authors determined the WBC thresholds for adult and pediatric AML patients as 75 × 10^9^/L and 165 × 10^9^/L, respectively, and reported that these thresholds would help identify more patients requiring immediate intervention. They also reported an association between HL and mutations in several genes including NPM1, FLT3, and DNMT3A. Differential gene expression and gene set enrichment analysis showed a decrease in the expression of genes involved in various cellular mechanisms, including cell adhesion, but an increase in the expression of genes in the mTOR signaling pathway. *In silico* analysis of drug sensitivity data revealed that inhibitors of BCL-2, histone deacetylase, and mTOR may be used for the treatment of AML with HL. Finally, they reported their clinical observations that the addition of a BCL-2 inhibitor to standard therapy decreases WBC counts, reduces tumor burden, and consequently relieves the symptoms.

Xue et al., retrospectively analyzed the data of pediatric BCP-ALL patients from Peking University People’s Hospital and investigated the prognostic role of WT1 expression level in these patients. The authors analyzed WT1 expression in ETV6-RUNX1, TCF3-PBX1, KMT2A-r, BCR-ABL1, and B-other groups in BCP-ALL patients and found that the WT1 level was higher in KMT2A-r than in the other groups. Researchers have also used X-tile, a useful tool to evaluate the biological association between a biomarker and clinical outcome, and divided all patients into two groups: WT1/ABL ≥0.24% (group A) and <0.24% (group B). Researchers have reported that the proportion of patients at high risk or with minimal residual disease (MRD) ≥0.01% at week 12 was higher in group A than in group B. They reported that although WT1 overexpression had lower 5 year OS and EFS rates in group A, it was not an independent risk factor. In contrast, in the B-other group, increased WT1 levels were an independent factor for lower OS and EFS and higher MRD ≥0.01% at week 12.

Heinz et al., conducted a pilot study in a small cohort to search for microbial and viral DNA using whole-metagenome shotgun sequencing of peripheral blood and bone marrow samples to test infection-related hypothesis in pediatric ALL. They comparatively studied treatment-naïve pediatric ALL patients and healthy children (or children with non-oncologic diseases) and reported no evidence of infectious DNA-based agents in the peripheral blood and bone marrow.

Chen et al., investigated the immune landscape within the tumor microenvironment of AML using bulk RNA sequencing data, with together somatic mutation and single-cell RNA data, and identified 2 sub-group according to their analysis results. In sub-group 1 samples, monocyte and macrophage infiltration was more common and a higher mutation ratio was reported in the FLT3, DNMT3A, and NPM1 genes by the authors. In addition, the prognosis of sub-group 1 AML patients was worse than that of sub-group 2. The authors also comparatively investigated AML T-cell-based immunotherapy target antigens between identified subgroups and reported that although CLEC12A, Folate receptor β, IL1RAP, and TIM3 expression was higher in sub-group 1, CD117, CD244, CD96, WT, and TERT levels were higher in sub-group 2. Finally, researchers showed that sub-group 1 was more sensitive to Elesklomol and Panobinostat and had a higher resistance to venetoclax than sub-group 2.

Klement and Drube, summarized and reviewed the studies addressing the relationship between FLT3 and CXCR4 in AML and provided an overview of these studies. The relationship between FLT3 and CXCR4 is interesting, but studies using different models may show conflicting results, as discussed by the authors. Indeed, several studies on patient samples have shown that FLT3-ITD expression leads to increased surface CXCR expression. However, *in vitro* studies have shown that FLT3-ITD decreases CXCR4 expression. Finally, as the authors stated, the relationship between FLT3 and CXCR in patient samples is difficult to reproduce fully under standard *in vitro* conditions, and systemic analyses are needed in model cell systems for this purpose.

As the guest editors of this research topic, we thank all the authors and reviewers who contributed.

